# A Comparative Assessment of Long-Term Care Financing and Service Delivery Models

**DOI:** 10.1093/geroni/igab046.073

**Published:** 2021-12-17

**Authors:** Larry Polivka, LuMarie Polivka-West

**Affiliations:** 1 Florida State University, Tallahassee, Florida, United States; 2 Florida State University, Florida State University, Florida, United States

## Abstract

The face of public long term care (LTC) funded largely through the Medicaid program is changing rapidly in the U.S. Over the last decade, most states have moved to managed LTC programs in various forms, with a growing number transferring all their programs, home and community based (HCBS) and nursing home services, to a Medicaid (MLTC) model. The amount of rigorously conducted and reported evaluation results on these programs are still very limited. Enough information is available, however, from other sources for at least preliminary comparison of relative cost-effectiveness of MLTC vs. traditional, non-profit models of public LTC services delivery and financing, as discussed in this paper. This comparison will show that, at this point, the MLTC programs are not more cost-effective than the traditional model of LTC administration. In fact, these initial assessments seem to indicate that the traditional model may be superior to the corporate for-profit MLTC model.

